# Transcriptome profiling identifies a recurrent *CRYL1-IFT88* chimeric transcript in hepatocellular carcinoma

**DOI:** 10.18632/oncotarget.17244

**Published:** 2017-04-19

**Authors:** Yi Huang, Jiaying Zheng, Dunyan Chen, Feng Li, Wenbing Wu, Xiaoli Huang, Yanan Wu, Yangyang Deng, Funan Qiu

**Affiliations:** ^1^ Provincial Clinical College, Fujian Medical University, Fuzhou 350001, Fujian, China; ^2^ Department of Clinical Laboratory, Fujian Provincial Hospital, Fuzhou 350001, Fujian, China; ^3^ Department of Pathology, Fujian Provincial Hospital, Fuzhou 350001, Fujian, China; ^4^ Department of Bioinformatics, MyGene Diagnostics Co., Ltd, Guangzhou 510300, Guangdong, China; ^5^ Department of Hepatobiliary Surgery, Fujian Provincial Hospital, Fuzhou 350001, Fujian, China

**Keywords:** HCC, transcriptome sequencing, fusion transcript, *CRYL1-IFT88*, tumorigenesis

## Abstract

We performed transcriptome sequencing for hepatocellular carcinoma (HCC) and adjacent non-tumorous tissues to investigate the molecular basis of HCC. Nine HCC patients were recruited and differentially expressed genes (DEGs) were identified. Candidate fusion transcripts were also identified. A total of 1943 DEGs were detected, including 690 up-regulated and 1253 down-regulated genes, and enriched in ten pathways including cell cycle, DNA replication, p53, complement and coagulation cascades, etc. Seven candidate fusion genes were detected and *CRYL1-IFT88* was successfully validated in the discovery sequencing sample and another 5 tumor samples with the recurrent rate of about 9.52% (6/63). The full length of *CRYL1-IFT88* was obtained by 3′ and 5′ RACE. The function of the fusion transcript is closed to *CRYL1* because it contained most of domain of *CRYL1*. According to the bioinformatics analysis, *IFT88*, reported as a tumor suppressor, might be seriously depressed in the tumor cell with this fusion because the transcript structure of *IFT88* was totally changed. The function depression of *IFT88* caused by gene fusion *CRYL1-IFT88* might be associated with tumorigenesis or development of HCC.

## INTRODUCTION

Hepatocellular carcinoma (HCC) is one of the most common malignancies worldwide [1–3]. About 745,000 deaths per year can be attributed to HCC [4]. Hepatic resection is currently the most optimal choice for HCC treatment. However, surgical resection is not applicable in most patients, and its long-term prognosis remains unsatisfactory [5]. To date, it is known that both cellular changes and etiological agents (i.e., virus infection and alcohol) are responsible for the cause of HCC [6, 7]. However, like any other complex diseases [8], the molecular pathogenesis of HCC remains poorly understood [9]. The lack of good diagnostic markers and therapeutic targets has rendered HCC a major challenge.

Recently, with dramatically increased throughput, next-generation sequencing provides an efficient tool to illustrate the transcriptome characteristics of cancers, including HCC. Transcriptome sequencing has been used to identify latent biomarkers for HCC [10–12], implicating its great potential in exploring the molecular basis of HCC. Of note, by using transcriptome sequencing, a recurrent chimeric transcript *DNAJB1-PRKACA* [13] was identified in fibrolamellar HCC (FL-HCC) patients, suggesting that this fusion transcript contributes to the pathogenesis of the FL-HCC and may represent a therapeutic target. Therefore, transcriptome sequencing is a revolutionary tool to investigate the cancer transcriptome and identify possible therapeutic targets [14].

In this study, we performed transcriptome sequencing for HCC and adjacent non-tumorous tissues to investigate the molecular basis of HCC. Nine patients diagnosed as primary HCC were recruited and differentially expressed genes (DEGs) were identified. Candidate fusion transcripts were also identified by using defuse [15]. Further RT-PCR and Sanger sequencing experiments were performed to validate potential recurrent fusion transcripts in other 54 pairs of tumor and adjacent non-tumor samples. Our investigation may shed light on the molecular event responsible for the progression of HCC and offer new possibilities for clinical management of HCC patients.

## RESULTS

### Overview of transcriptome sequencing statistics

Pair-end second-generation transcriptome sequencing was performed in nine HCC patients. Sample characteristics are list in Table 1. An average of 35,772,695 pair-end 125 bp clean reads was generated (Table 2). The average mapping rate was 93.17%, resulting an average coverage of depth of 32x (Table 2).

**Table 1:** Sample characteristics

Patient	Age	Gender	Hepatitis	Serum AFP level(ng/mL)	Metastasis	Glisson capsule invasion	Tumor size (mm)	Multiple liver nodules
***P10***	***59***	***M***	***HBV***	***670.10***	***No***	***–***	***35***	***-***
**P14**	**50**	**M**	**HBV**	**12483**	**No**	**+**	**50**	**-**
**P17**	**37**	**M**	**HBV**	**4.47**	**No**	**+**	**41**	**-**
**P21**	**62**	**M**	**HBV**	**266.5**	**No**	**+**	**32**	**-**
**P24**	**76**	**M**	**HCV**	**5.84**	**No**	**+**	**70**	**-**
**P26**	**59**	**M**	**HBV**	**3.73**	**No**	***–***	**6**	**-**
**P29**	**69**	**M**	**HBV**	**6.90**	**No**	**+**	**160**	**synchronous**
**P36**	**61**	**M**	**NBNC**	**1.11**	**No**	**+**	**80**	**-**
**P40**	**19**	**F**	**NBNC**	**2.85**	**No**	***–***	**29**	**-**
*L24*	*63*	*M*	*HBV*	*2975*	*No*	*+*	*70*	*synchronous*
*L26*	*47*	*M*	*HBV*	*5375*	*Yes*	*+*	*130*	*-*
*L44*	*72*	*M*	*HBV*	*303.8*	*No*	*+*	*40*	*synchronous*
*L134*	*66*	*M*	*HBV*	*60500*	*No*	*+*	*50*	*-*
*H19*	*59*	*M*	*NBNC*	*54.84*	*No*	*+*	*26*	*-*
L6	48	M	NBNC	55.1	Yes	+	180	-
L21	41	M	HBV	4.70	Yes	+	50	-
L25	72	M	HBV	1110	No	+	80	-
L30	50	M	HBV	260.9	No	***–***	25	-
L36	64	M	HBV	244.6	Yes	+	65	synchronous
L39	67	M	HBV	321.1	No	+	55	synchronous
L45	71	M	HBV	4606	Yes	+	12	-
L46	18	F	HBV	37979	No	+	70	-
L49	45	M	HBV	6528	No	+	60	synchronous
L52	50	M	HBV	3.03	Yes	+	55	synchronous
L53	61	M	HBV	1.90	No	+	30	-
L54	52	M	HBV	26.53	No	+	20	-
L55	55	M	HBV	1.82	No	+	34	-
L57	58	F	HBV	2.55	Yes	+	55	synchronous
L61	42	M	HBV	3.38	No	+	20	synchronous
L64	64	M	HBV	774.2	No	+	10	-
L68	54	F	NBNC	23784	No	+	22	-
L72	40	M	HBV	1816	No	+	30	-
L73	74	M	NBNC	5.90	No	+	30	-
L85	76	M	NBNC	2.10	No	+	34	-
L154	47	M	HBV	62.47	Yes	+	105	-
L195	67	M	NBNC	3.01	Yes	+	80	-
H1	37	M	HBV	1810	No	***–***	55	-
H2	67	M	HBV	3103	No	+	40	-
H3	48	M	HBV	2.07	No	***–***	35	-
H4	68	M	HBV	15.87	No	+	35	-
H5	71	M	HBV	11.65	No	+	35	-
H6	56	M	HBV	33571	No	+	57	-
H7	52	M	HBV	2100	Yes	***–***	35	-
H8	52	M	NBNC	5.30	No	+	65	-
H9	37	M	HBV	>60500	Yes	***–***	95	-
H11	46	M	HBV	4.64	No	***–***	45	-
H13	52	F	NBNC	2.45	No	+	22	-
H16	75	M	NBNC	4.14	Yes	+	87	synchronous
H22	47	M	HBV	5.78	No	+	40	-
H25	60	M	HBV	61.54	No	***–***	25	-
H27	65	M	HBV	5.34	No	+	70	-
H28	63	M	HBV	3396	No	+	40	-
H29	52	M	HBV	66.50	No	+	22	-
H30	58	M	HBV	6.52	Yes	+	58	synchronous
H31	42	M	HBV	3.31	No	+	30	-
H32	62	M	HBV	570	No	+	28	-
H33	46	M	HBV	3780	No	+	21	synchronous
H34	64	M	HBV	36541	Yes	+	140	-
H35	47	F	HCV	250.5	No	***–***	22	-
H36	39	M	HBV	8.10	Yes	+	22	synchronous
H37	63	M	HBV	208	No	+	40	-
H38	48	M	NBNC	3.07	No	+	33	-
H39	66	M	HBV	82.81	No	+	34	-

Note: The nine samples used for sequencing are shown in bold. The six samples with validated fusion *CRYL1*-*IFT88* are shown in italic.

**Table 2:** Summary statistics of the transcriptome sequencing

Patient	Sample type	Total reads	Mapped reads	Total base (bp)	Mapped base (bp)	Mappping ratio	Coverage (X)
P10	T	35,930,408	33,819,287	4,491,301,000	4,227,410,875	94.12%	33
	N	34,902,228	32,863,973	4,362,778,500	4,107,996,625	94.16%	32
P14	T	35,315,378	33,125,921	4,414,422,250	4,140,740,125	93.80%	32
	N	34,383,802	32,279,443	4,297,975,250	4,034,930,375	93.88%	31
P17	T	35,532,658	33,258,212	4,441,582,250	4,157,276,500	93.60%	32
	N	34,537,914	32,152,860	4,317,239,250	4,019,107,500	93.09%	31
P21	T	34,762,386	32,701,011	4,345,298,250	4,087,626,375	94.07%	31
	N	35,314,918	33,021,170	4,414,364,750	4,127,646,250	93.50%	32
P24	T	35,610,444	32,509,118	4,451,305,500	4,063,639,750	91.29%	31
	N	34,637,398	31,280,461	4,329,674,750	3,910,057,625	90.31%	30
P26	T	34,566,526	32,670,216	4,320,815,750	4,083,777,000	94.51%	31
	N	35,384,486	33,407,169	4,423,060,750	4,175,896,125	94.41%	32
P29	T	34,564,642	32,385,294	4,320,580,250	4,048,161,750	93.69%	31
	N	34,771,048	32,792,475	4,346,381,000	4,099,059,375	94.31%	32
P36	T	35,371,920	32,571,695	4,421,490,000	4,071,461,875	92.08%	31
	N	35,959,532	32,990,753	4,494,941,500	4,123,844,125	91.74%	32
P40	T	42,279,434	39,097,967	5,284,929,250	4,887,245,875	92.50%	38
	N	40,083,384	36,852,292	5,010,423,000	4,606,536,500	91.90%	35
Average		35,772,695	33,321,073	4,471,586,847	4,165,134,146	93.17%	32
Total		643,908,506	599,779,317	80,488,563,250	74,972,414,625		

### DEGs analyses results

We next detected DEGs between tumor and non-tumor samples. A total of 1943 DEGs were detected, including 690 up-regulated and 1253 down-regulated genes.

DEGs were subjected to KEGG pathway analyses. As shown in Table 3, with the cut off of FDR < 0.05, DEGs were enriched in ten pathways, including cell cycle (hsa04110), DNA replication (hsa03030), p53 (hsa04115) and complement and coagulation cascades pathway (hsa04610), as well as retinol, xenobiotics by cytochrome P450, drug, arachidonic acid, tyrosine and fatty acid metabolism pathway (hsa00830, 00980, 00982, 00590, 00350, 00071). The GO category enrichment analyses resulted extensive items overrepresented with DEGs. As shown in Table 3, GO items (FDR < 10^–3^) enriched with DEGs were associated with cell cycle and other processes, such as immune response, DNA replication, complement activation, oxidation, metabolism, and so on.

**Table 3:** KEGG pathway and gene ontology biological process items enrichment analyses result for the DEGs

Term	DEG Count	FDR
KEGG pathway		
hsa04110:Cell cycle	31	6.10 × 10^–6^
hsa00830:Retinol metabolism	17	1.82 × 10^–4^
hsa00980:Metabolism of xenobiotics by cytochrome P450	17	7.69 × 10^–4^
hsa03030:DNA replication	12	2.97 × 10^–3^
hsa00982:Drug metabolism	16	4.21 × 10^–3^
hsa04610:Complement and coagulation cascades	15	9.85 × 10^–3^
hsa00590:Arachidonic acid metabolism	13	1.03 × 10^–2^
hsa00350:Tyrosine metabolism	11	3.96 × 10^–2^
hsa00071: Fatty acid metabolism	10	4.17 × 10^–2^
hsa04115:p53 signaling pathway	13	4.58 × 10^–2^
GO item		
GO:0022403~cell cycle phase	84	5.15 × 10^–16^
GO:0000278~mitotic cell cycle	77	4.11 × 10^–15^
GO:0000279~M phase	70	3.45 × 10^–14^
GO:0007067~mitosis	54	1.87 × 10^–13^
GO:0000280~nuclear division	54	1.87 × 10^–13^
GO:0048285~organelle fission	55	2.72 × 10^–13^
GO:0000087~M phase of mitotic cell cycle	54	4.11 × 10^–13^
GO:0007049~cell cycle	118	1.26 × 10^–12^
GO:0022402~cell cycle process	94	3.43 × 10^–12^
GO:0009611~response to wounding	87	6.60 × 10^–11^
GO:0006954~inflammatory response	61	7.36 × 10^–10^
GO:0051301~cell division	57	1.09 × 10^–9^
GO:0007059~chromosome segregation	26	8.40 × 10^–9^
GO:0006952~defense response	90	1.38 × 10^–8^
GO:0006955~immune response	97	2.41 × 10^–8^
GO:0051726~regulation of cell cycle	53	4.06 × 10^–6^
GO:0006260~DNA replication	36	9.33 × 10^–6^
GO:0002526~acute inflammatory response	24	1.13 × 10^–5^
GO:0002253~activation of immune response	23	2.07 × 10^–5^
GO:0050778~positive regulation of immune response	29	4.88 × 10^–5^
GO:0007051~spindle organization	15	5.21 × 10^–5^
GO:0031960~response to corticosteroid stimulus	21	5.78 × 10^–5^
GO:0051384~response to glucocorticoid stimulus	20	5.84 × 10^–5^
GO:0048545~response to steroid hormone stimulus	34	9.52 × 10^–5^
GO:0055114~oxidation reduction	79	1.70 × 10^–4^
GO:0010033~response to organic substance	86	2.60 × 10^–4^
GO:0002684~positive regulation of immune system process	38	2.84 × 10^–4^
GO:0002252~immune effector process	26	3.08 × 10^–4^
GO:0051329~interphase of mitotic cell cycle	22	3.60 × 10^–4^
GO:0045087~innate immune response	26	5.19 × 10^–4^
GO:0000079~regulation of cyclin-dependent protein kinase activity	15	5.37 × 10^–4^
GO:0000226~microtubule cytoskeleton organization	27	5.58 × 10^–4^
GO:0051325~interphase	22	5.67 × 10^–4^
GO:0006956~complement activation	13	6.96 × 10^–4^
GO:0008203~cholesterol metabolic process	20	7.33 × 10^–4^
GO:0000070~mitotic sister chromatid segregation	12	7.35 × 10^–4^
GO:0009719~response to endogenous stimulus	54	7.58 × 10^–4^
GO:0000819~sister chromatid segregation	12	9.75 × 10^–4^

### Fusion genes detection and validation

**Figure 1:**
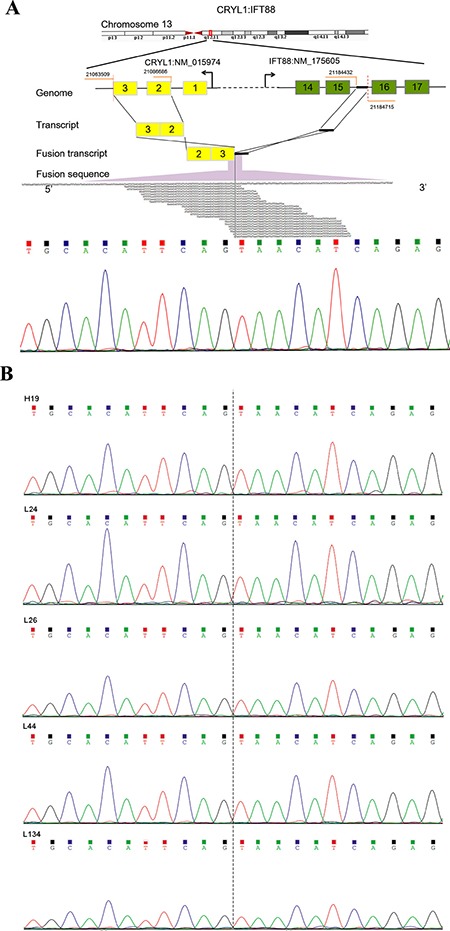
Detection and Sanger sequencing validation of a recurrent fusion transcript *CRYL1-IFT88* (**A**) Illustration of the *CRYL1*-*IFT88* fusion gene (top) and Sanger sequencing validation result for the transcriptome tumor sequencing sample (P10) (bottom). (**B**) Sanger sequencing validation result for the other five tumor samples. The IDs of samples corresponded to Table 1. The fusion point was marked with dash line.

**Figure 2:**
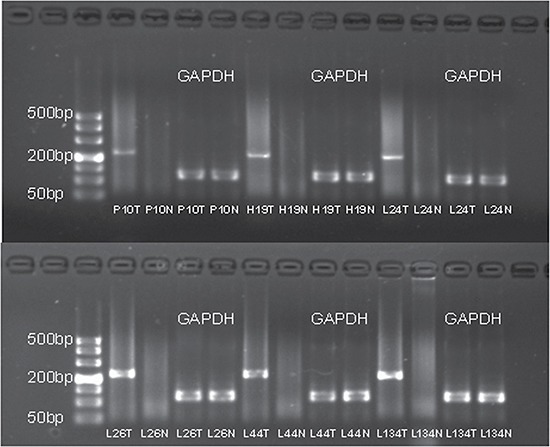
The agarose gel electrophoresis of the six samples with successfully validated fusion transcript *CRYL1-IFT88* The RT-PCR product for the fusion gene was 230 bp. The IDs of samples corresponded to Table 1. T and N represented tumor and adjacent non-tumor tissue, respectively.

**Figure 3:**
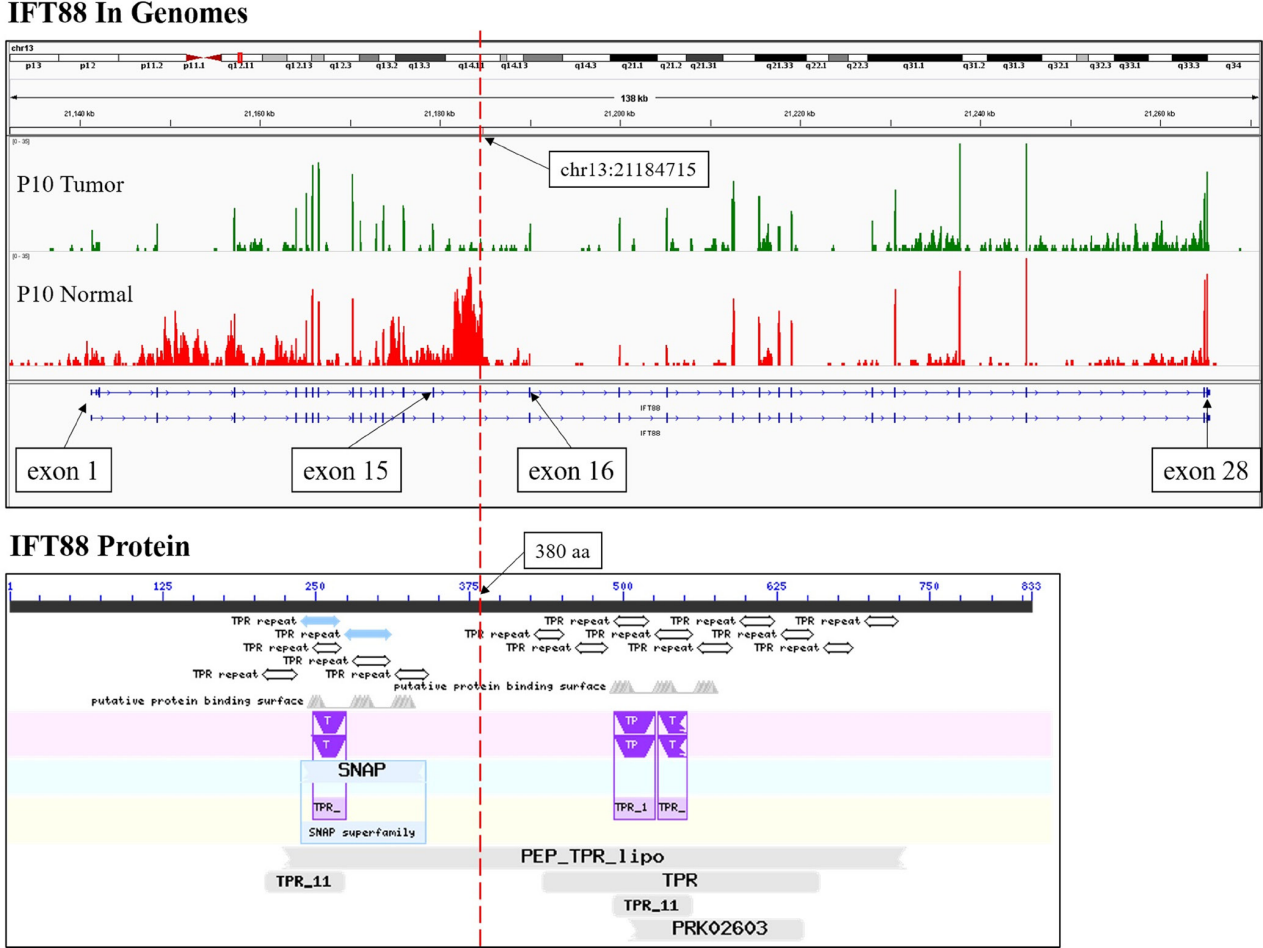
The RNA-Seq reads coverage and functional domains of IFT88 The RNA-Seq reads coverage of gene *IFT88* by IGV in the samples of patient P10 was shown at the top, and the functional domains of IFT88 according to NCBI CD-search was shown at the bottom. The red dotted line indicated the breakpoint.

To detect fusion transcripts that might be the potential cause for tumorigenesis, the software defuse was used and after the filtering process as described in the methods section. Seven candidate fusion genes were detected (Table 4). Using RT-PCR and Sanger sequencing, the fusion gene *CRYL1*-*IFT88* was successfully validated in the discovery sequencing sample (P10 tumor sample) and it was also validated in another 5 tumor samples (Figures 1, 2). Therefore, this fusion gene is considered as a recurrent fusion transcript related to HCC with the recurrent rate of about 9.52% (6/63). To investigate how the gene fusion *CRYL1*-*IFT88* affected their expression and proteins, we observed the reads coverage in genomes by IGV (Integrative Genomics Viewer) and domains by NCBI CD-search (Conserved Domains search). We found that quite a lot RNA-Seq reads covered in the intron between exon 15 and exon 16 of *IFT88* in tumor sample of P10, but this was not observed in the matched normal sample (Figure 3 top), which indicated that the transcription structure of *IFT88* was very likely changed in the tumor cells with this fusion. This break was just occurred in the middle of IFT88 protein sequence ((Figure 3 bottom), which might totally destroy the function of IFT88. However, the change of reads coverage was not observed in the genes *CRYL1*, it might be the high expression of wild-type *CRYL1* masked the change of expression structure.

**Table 4:** Information of the identified fusion genes

Sample id	Sample type	Gene1-Gene2	Break point1	Break point2	Break point1 location	Break point2 location
P10	tumor	*CRYL1*-*IFT88*	chr13:21063509	chr13:21184715	coding	intron
P14	tumor	*BRD9-OR4*N2	chr5:886707	chr14:20230047	coding	5′-UTR
P14	tumor	*MPV17*-*TRIM54*	chr2:27545315	chr2:27527827	coding	coding
P24	tumor	*BHMT-MTATP6P1*	chr5:78427385	chr1:569753	3′-UTR	exon
P26	tumor	*NOMO3-XYLT1*	chr16:16372646	chr16:17323802	coding	intron
P26	tumor	*NOMO3-XYLT1*	chr16:16372646	chr16:17318037	coding	intron
P29	tumor	*TNFSF14-C3*	chr19:6664861	chr19:6677181	3′-UTR	downstream

To discover the function of the fusion transcript, we performed both 3′ and 5′ RACE (rapid-amplification of cDNA ends) experiments and obtained the full-length *CRYL1-IFT88* fusion sequence (779 bp). The longest ORF was 279 bp (predicted by NCBI ORF-finder) and corresponding to a 92 aa protein sequence (see Supplementary File.docx). And the fusion protein contained most of the domain of CRYL1, including 3-hydroxybutyryl-CoA dehydrogenase domain, NAD binding domain, and so on, which indicated that the functional of the CRYL1-IFT88 were similar with CRYL1 (see Supplementary File.docx).

## DISCUSSION

We have applied the transcriptome sequencing approach to illustrate the gene expression characteristics of HCC. Pathway analyses showed that ten pathways, including cell cycle, DNA replication, p53 and complement and coagulation cascades as well as six metabolism processes, were overrepresented with DEGs. Deregulation of the cell cycle [16], DNA replication [17] and p53 pathways [18] are expected since uncontrolled cell division and aberrant tumor suppressor are the major character of cancer cells. As for the complement and coagulation cascades pathway, consistent with our results, both gene expression [19] and proteomics [20] analyses have shown that this pathway is related to the pathogenesis of HCC.

Gene fusion is an important event involved in the development of various types of malignancies, which is the consequence of the genomic rearrangements with a deletion, insertion, translocation or inversion of distal intra- or inter-chromosomal sequences [21]. The oncogenic activation may be triggered by the gene activation or repression due to gene fusion. The Philadelphia chromosome found in chronic myelogenous leukemia (CML) consisting of the BCR-ABL fusion gene is a classical example of gene fusion, an activated tyrosine kinase that drives CML [22]. Recent technology advances especially transcriptome sequencing and bioinformatics strategy for processing cancer profiling data, make the discovery of more fusion genes in cancers, including a recurrent chimeric transcript *DNAJB1-PRKACA* in FL-HCC [9] and *SLC45A3-ELK4* in prostate cancers [23], etc. These known fusion genes provide key insights into tumor biology and have significant clinical impact by serving as potential diagnostic markers or therapeutic targets. In our study, we detected seven chimeric transcripts in total by transcriptome sequencing. Fusion genes were subjected to validation and successfully confirmed that *CRYL1*-*IFT88* is a recurrent fusion transcript related with HCC. To our knowledge, this fusion gene was first reported and validated in HCC. Protein encoded by *CRYL1* catalyzes the dehydrogenation of L-gulonate into dehydro-L-gulonate in the uronate cycle, which is an alternative glucose metabolic pathway. Reduced expression of *CRYL1* in HCC has been observed in many studies [24–26]. Moreover, it was reported that reduced *CRYL1* expression in HCC confers cell growth advantages and correlates with adverse patient prognosis [26]. Protein encoded by *IFT88* is a member of the tetratrico peptide repeat (TPR) family, and is involved in liver oval cell proliferation, differentiation, and ploidy control [27]. Tumor suppression activity of this gene was demonstrated and this gene was reported as a liver neoplasia tumor suppressor gene in a previous study [28]. According to our bioinformatics analysis, the protein function of IFT88 might be suppressed due to the gene fusion. Considering the potential involvement of these two genes in HCC, the fusion transcript we identified here might be responsible for the tumorigenesis and serve as potential targets for further therapeutic strategy by the overexpression of *IFT88* to overcome the function inhibition due to gene fusion *CRYL1-IFT88*.

In conclusion, we used transcriptome sequencing approach to illustrate the gene expression characteristics of HCC. Cell cycle, DNA replication, p53 and complement and coagulation cascades pathways as well as some metabolism processes were overrepresented with DEGs. Of note, we detected and successfully validated *CRYL1*-*IFT88* as a recurrent fusion transcript in HCC with the recurrent rate of about 9.52% (6/63).

## MATERIALS AND METHODS

### Ethics statement

Our study design was approved by the institutional review board of the Fujian provincial hospital. Written informed consent was obtained from all subjects.

### Subjects

Sixty-three subjects aged from 18 to 76 were diagnosed as primary HCC in the Fujian provincial hospital during the period from 03/01/2014 to 12/31/2015. Hepatitis B virus (HBV) related tumors were defined according to the presence of HB surface antigen (HBsAg) in serum, and hepatitis C virus (HCV) related tumors were according to the presence of antibody to HCV (HCVAb) in serum. NBNC tumor was defined according to the absence of both HBsAg and HCVAb in serum. Primary tumor and adjacent non-tumorous samples were obtained from all patients who underwent surgical tumor resection. All samples were frozen immediately at –80°C until RNA extraction. Total RNA was isolated by using RecoverAll™ Total Nucleic Acid Isolation Kit (Life Technologies, Carlsbad, CA, USA). Integrity of RNA was assessed by Agilent 2100 bioanalyzer (Agilent, Santa Clara, CA, USA). RNA from nine samples was subjected to sequencing and other samples were used in the validation experiments.

### Transcriptome sequencing

Sequencing libraries were prepared by using prepared by using TruSeq RNA Sample Prep Kit (Illumina, San Diego, CA, USA) according to standard protocols. Briefly, total RNA was firstly randomly fragmented and poly-A-selected. Secondly, the RNA fragments were reverse transcribed to cDNA, end-repaired and ligated with adapters. The libraries then underwent size selection, PCR and purification. The quality of libraries was assessed by using Bioanalyzer 2100 (Agilent, Santa Clara, CA, USA). Sequencing was then performed on an Illumina HiSeq 2500 sequencer with 125 bp pair-end reads. All raw data have been deposited in the NIH Short Read Archive database (Access number: SRP 102722).

### Reads processing

Raw sequencing reads were firstly filtered for adapters and ribosomal RNA. Reads containing five or more low quality (quality score < 20) bases were also removed. The remained high-quality reads were then aligned to human genome (hg19) by using Tophat [29]. The mapped reads were then subjected to alignment against the the human transcriptome (Ensembl, GRCh37.73). Gene expression level measured by FPKM (fragments per kilobase per million) was calculated by Cufflinks [30]. All processed expression data have been submitted to GEO database (Access number: GSE 97214).

### Differentially expressed genes (DEGs) analysis

For each group, DEGs between the tumor and matched non-tumor tissues were selected with pair-wise *t* test and the significant threshold was set as *p-value* of less than 0.05 and |log_2_(fold change, FC)| > = 1. DAVID [31] was used to do the Gene Ontology (GO) and KEGG pathway annotation and enrichment analyses. The significant threshold for enrichment was set as false discovery rate (FDR) < 0.05.

### Fusion gene detection

Fusion transcripts were identified by defuse [15]. The default filtering processes of defuse were carried out as previously described [15]. The results of deFuse were further filtered to reduce false positives with the following criteria: 1) predictions supported by less than eight reads were removed; 2) predictions between adjacent genes were filtered unless implied in genomic inversion or eversion; 3) predictions related to ribosomal proteins or small nuclear ribosomal proteins were removed.

### Fusion gene validation

Selected fusion transcripts were subjected to validation using RT-PCR and Sanger sequencing. For the RT-PCR reactions, total RNA was converted to cDNA with random hexamer primers using the High-Capacity cDNA Reverse Transcription kit (Applied Biosystems, Foster City, CA, USA). The RT-PCR products were gel purified and sequenced by Sanger sequencing using an ABI 3730 DNA Sequencer (Applied Biosystems, Foster City, CA, USA).

### 5′ RACE and 3′ RACE

5′ RACE. RLM-RACE was performed with the SMARTer™ RACE cDNA Amplification Kit (Clontech). Total RNA (10 mg) was first treated with calf intestinal alkaline phosphatase (CIP) to remove 5′ phosphate groups, followed by tobacco acid pyrophosphatase to remove 5′ cap structures. After RNA linker ligation, mRNA transcripts were reverse-transcribed with SMARTScribe™ reverse transcriptase. To amplify first-strand cDNAs, we performed NEST PCR. Firstly, outer 5′ PCR using 5′ RACE outer primers (provided in kit) and a *IFT88* intron 15 primer (TAGGGAATGACAGGAAACGGGGAT) with SuperTaq Plus polymerase (Applied Biosystems). Subsequently, inner 5′ PCR was performed with a 5′ RACE inner primer (provided in kit) and a *CYRL1* exon 3 primer (TTCCA CACTCAGGGAGCCTTTCA). After gel electrophoresis, PCR bands of interest were excised and cloned. The PCR production were sequenced bidirectionally on an ABI 3730 automated sequencer (Applied Biosystems). A minimum of 3 independent colonies were sequenced in each experiment.

### 3′ RACE

RLM-RACE was performed with the SMARTer™ RACE cDNA Amplification Kit (Clontech). Total RNA (10 mg) was first treated with calf intestinal alkaline phosphatase (CIP) to remove 3′ phosphate groups, followed by tobacco acid pyrophosphatase to remove 3′ cap structures. After RNA linker ligation, mRNA transcripts were reverse-transcribed with SMARTScribe™ reverse transcriptase. To amplify first-strand cDNAs, we performed NEST PCR, firstly, outer 3′ PCR using 3′ RACE outer primers (provided in kit) and a *CYRL1* exon 2 primer (GAGGCTTCCAGGTGAAACTCTATGA) with SuperTaq Plus polymerase (Applied Biosystems). Subsequently, inner 3′ PCR was performed with a 3′ RACE inner primer (provided in kit) and a *IFT88* intron 15 primer (TTATCC CCGTTTCCTGTCATTCCCT). After gel electrophoresis, PCR bands of interest were excised and cloned. The PCR production were sequenced bidirectionally on an ABI 3730 automated sequencer (Applied Biosystems). A minimum of 3 independent colonies were sequenced in each experiment.

## SUPPLEMENTARY MATERIALS



## Abbreviations

HCC: (hepatocellular carcinoma)
DEGs: (differentially expressed genes) RACE (Rapid-Amplification of cDNA Ends)
RLM-RACE: (RNA Ligase-Mediated-Rapid Amplification of cDNA Ends)
FL-HCC: (fibrolamellar hepatocellular carcinoma)
GO enrichment analyses: (Gene Ontology-based statistical enrichment analysis)
FDR: (false discovery rate)
IGV: (Integrative Genomics Viewer)
CD-search: (Conserved Domains search)
ORF: (open reading frame)
NAD: (Nicotinamide adenine dinucleotide)
TPR: (tetratrico peptide repeat)
HBV: (Hepatitis B virus)
HBsAg: (HB surface antigen)
HCV: (Hepatitis C virus)
NBNC tumor: (non-B non-C tumor)
FPKM: (fragments per kilobase per million)
CIP: (calf intestinal alkaline phosphatase)
CRYL1: (crystallin, lambda 1)
IFT88: (intraflagellar transport 88)
BRD9: (bromodomain containing 9)
OR4N2: (olfactory receptor, family 4, subfamily N, member 2)
MPV17: (MpV17 mitochondrial inner membrane protein)
TRIM54: (tripartite motif containing 54)
BHMT: (betaine-homocysteine S-methyltransferase)
MTATP6P1: (mitochondrially encoded ATP synthase 6 pseudogene 1)
NOMO3: (NODAL modulator 3)
XYLT1: (xylosyltransferase I)
TNFSF14: (tumor necrosis factor (ligand) superfamily, member 14)
C3: (complement component 3)

## References

[1] Colombo M (1992). Hepatocellular carcinoma. Journal of hepatology.

[2] Raza A, Sood GK (2014). Hepatocellular carcinoma review: current treatment, and evidence-based medicine. World journal of gastroenterology.

[3] He B, Qiu X, Li P, Wang L, Lv Q, Shi T (2010). HCCNet: an integrated network database of hepatocellular carcinoma. Cell Res.

[4] Cancer Organization WH (2015).

[5] Fan ST, Mau Lo C, Poon RT, Yeung C, Leung Liu C, Yuen WK, Ming Lam C, Ng KK, Ching Chan S (2011). Continuous improvement of survival outcomes of resection of hepatocellular carcinoma: a 20-year experience. Annals of surgery.

[6] He B, Zhang H, Shi T (2011). A comprehensive analysis of the dynamic biological networks in HCV induced hepatocarcinogenesis. PLoS One.

[7] He B, Li T, Guan L, Liu FE, Chen XM, Zhao J, Lin S, Liu ZZ, Zhang HQ (2016). CTNNA3 is a tumor suppressor in hepatocellular carcinomas and is inhibited by miR-425. Oncotarget.

[8] He B, Lu C, Wang M, Zheng G, Chen G, Jiang M, He X, Bian Z, Zhang G, Lu A (2015). Drug discovery in traditional Chinese medicine: From herbal fufang to combinatory drugs. Science.

[9] Bruix J, Boix L, Sala M, Llovet JM (2004). Focus on hepatocellular carcinoma. Cancer cell.

[10] Simon EP, Freije CA, Farber BA, Lalazar G, Darcy DG, Honeyman JN, Chiaroni-Clarke R, Dill BD, Molina H, Bhanot UK, MP La Quaglia, Rosenberg BR, Simon SM (2015). Transcriptomic characterization of fibrolamellar hepatocellular carcinoma.

[11] Ho DW, Kai AK, Ng IO (2015). TCGA whole-transcriptome sequencing data reveals significantly dysregulated genes and signaling pathways in hepatocellular carcinoma. Frontiers of medicine.

[12] Lin KT, Shann YJ, Chau GY, Hsu CN, Huang CY (2014). Identification of latent biomarkers in hepatocellular carcinoma by ultra-deep whole-transcriptome sequencing. Oncogene.

[13] Honeyman JN, Simon EP, Robine N, Chiaroni-Clarke R, Darcy DG, Lim II, Gleason CE, Murphy JM, Rosenberg BR, Teegan L, Takacs CN, Botero S, Belote R (2014). Detection of a recurrent DNAJB1-PRKACA chimeric transcript in fibrolamellar hepatocellular carcinoma. Science.

[14] Wang Z, Gerstein M, Snyder M (2009). RNA-Seq: a revolutionary tool for transcriptomics. Nature reviews Genetics.

[15] McPherson A, Hormozdiari F, Zayed A, Giuliany R, Ha G, Sun MG, Griffith M, Heravi Moussavi A, Senz J, Melnyk N, Pacheco M, Marra MA, Hirst M (2011). deFuse: an algorithm for gene fusion discovery in tumor RNA-Seq data. PLoS computational biology.

[16] Visconti R, Della Monica R, Grieco D (2016). Cell cycle checkpoint in cancer: a therapeutically targetable double-edged sword. J Exp Clin Cancer Res.

[17] Wasylishen AR, Lozano G (2016). Attenuating the p53 Pathway in Human Cancers: Many Means to the Same End. Cold Spring Harb Perspect Med.

[18] Vassilev A, DePamphilis ML (2017). Links between DNA Replication, Stem Cells and Cancer. Genes (Basel).

[19] Zhang L, Guo Y, Li B, Qu J, Zang C, Li F, Wang Y, Pang H, Li S, Liu Q (2013). Identification of biomarkers for hepatocellular carcinoma using network-based bioinformatics methods. European journal of medical research.

[20] Tsai TH, Song E, Zhu R, Di Poto C, Wang M, Luo Y, Varghese RS, Tadesse MG, Ziada DH, Desai CS, Shetty K, Mechref Y, Ressom HW (2015). LC-MS/MS-based serum proteomics for identification of candidate biomarkers for hepatocellular carcinoma. Proteomics.

[21] Heim S, Mitelman F (2008). Molecular screening for new fusion genes in cancer. Nature genetics.

[22] Nowell PC, Hungerford DA (1961). Chromosome studies in human leukemia. II. Chronic granulocytic leukemia. Journal of the National Cancer Institute.

[23] Rickman DS, Pflueger D, Moss B, VanDoren VE, Chen CX, de la Taille A, Kuefer R, Tewari AK, Setlur SR, Demichelis F, Rubin MA (2009). SLC45A3-ELK4 is a novel and frequent erythroblast transformation-specific fusion transcript in prostate cancer. Cancer research.

[24] Chen J, Yu L, Li D, Gao Q, Wang J, Huang X, Bi G, Wu H, Zhao S (2003). Human CRYL1, a novel enzyme-crystallin overexpressed in liver and kidney and downregulated in 58% of liver cancer tissues from 60 Chinese patients, and four new homologs from other mammalians. Gene.

[25] Chen CF, Yeh SH, Chen DS, Chen PJ, Jou YS (2005). Molecular genetic evidence supporting a novel human hepatocellular carcinoma tumor suppressor locus at 13q12.11. Genes, chromosomes & cancer.

[26] Cheng IK, Ching AK, Chan TC, Chan AW, Wong CK, Choy KW, Kwan M, Lai PB, Wong N (2010). Reduced CRYL1 expression in hepatocellular carcinoma confers cell growth advantages and correlates with adverse patient prognosis. The Journal of pathology.

[27] Richards WG, Yoder BK, Isfort RJ, Detilleux PG, Foster C, Neilsen N, Woychik RP, Wilkinson JE (1997). Isolation and characterization of liver epithelial cell lines from wild-type and mutant TgN737Rpw mice. The American journal of pathology.

[28] Isfort RJ, Cody DB, Doersen CJ, Richards WG, Yoder BK, Wilkinson JE, Kier LD, Jirtle RL, Isenberg JS, Klounig JE, Woychik RP (1997). The tetratricopeptide repeat containing Tg737 gene is a liver neoplasia tumor suppressor gene. Oncogene.

[29] Trapnell C, Pachter L, Salzberg SL (2009). TopHat: discovering splice junctions with RNA-Seq. Bioinformatics.

[30] Trapnell C, Williams BA, Pertea G, Mortazavi A, Kwan G, van Baren MJ, Salzberg SL, Wold BJ, Pachter L (2010). Transcript assembly and quantification by RNA-Seq reveals unannotated transcripts and isoform switching during cell differentiation. Nature biotechnology.

[31] Huang da W, Sherman BT, Lempicki RA (2009). Systematic and integrative analysis of large gene lists using DAVID bioinformatics resources. Nat Protoc.

