# Supplementary material for: Transcriptome profiling identifies a recurrent CRYL1-IFT88 chimeric transcript in hepatocellular carcinoma
Source: Oncotarget. 2017 Apr 19;8(25):40693–704. doi: 10.18632/oncotarget.17244 (PMC5522265; doi:10.18632/oncotarget.17244)

Transcriptome profiling identifies a recurrent *CRYL1-IFT88* chimeric transcript in hepatocellular carcinoma

Supplementary Materials

Supplementary File: the full-length CRYL1-IFT88 fusion sequence obtained by 3′ and 5′ RACE experiments

The full-length CRYL1-IFT88 fusion transcript (779 bp)

> CRYL1-IFT88

**GGCGCCGCGTCCCCGGCCCAACC<u>ATG</u>GC
GTCCTCCGCGGCCGGCTGCGTGGTGATCGTTG
GCAGTGGAGTCATTGGGCGAAGCTGGGCCATG
CTGTTTGCCAGTGGAGGCTTCCAGGTGAAACT
CTATGACATTGAGCAACAGCAGATAAGGAACG
CCCTGGAAAACATCAGAAAGGAGATGAAGTTG
CTGGAGCAGGCAGGTTCTCTGAAAGGCTCCCT
GAGTGTGGAAGAGCAGCTGTCACTCATCAGTG
GTTGTCCCAATATCCAAGAAGCAGTAGAGGGT
GCCATGCACATTCAG**|<u>TAA</u>CATCAGAGATTTCATT
AAACCTGGAGGTTGAATTCATTTCGGAGTTCAAT
GATATGTCCAAAGAATCAGGTACTATAATCGTATAG
ACAACTCCTGTAATATATCCACAGTGCAAAATTAT
CCCCGTTTCCTGTCATTCCCTAGTGAGAAAAATCC
AACTGCAGACTTGACTGCCTTCGGTGAAGGCACA
CGGCAAATTCTTCTGAAAAATAATACCCTTCAAGA
TTGACTCTTCCCGTTTTACACAGCAATTGTCTCAG
CTTGGGACCAAAGTATTTACAAAGCAAGCAAACA
ACAGACTAGCGAACAGGGACACTTCTGAAGCTGG
TGATGCAGAAATCCACTGCCAAGCAGGGTGGGGG
GCCAGTCACCTCTCCTGGAATGTTCCATATGTAAG
CTGACACAACTTAACATCTTAAATACAGAATTCCA
ATCAAAAGAAAAGCAAAAAAAAAAAAAAAAAAA
AAAAAAAAAAA

Note: Bold sequence was from CRYL1, and light sequence was from ITF88. The red sequence was the primer of 5′ and 3′ RACE experiments. Underline <u>ATG</u> and <u>TAA</u> were the start codon and stop codon. The breakpoint occurred in |<u>TAA</u>

The longest ORF predicted by NCBI ORF-finder (279 bp)

> CRYL1-IFT88 ORF

<u>ATG</u>GCGTCCTCCGCGGCCGGCTGCGTGGTGA
TCGTTGGCAGTGGAGTCATTGGGCGAAGCTGGGC
CATGCTGTTTGCCAGTGGAGGCTTCCAGGTGAAA
CTCTATGACATTGAGCAACAGCAGATAAGGAACG
CCCTGGAAAACATCAGAAAGGAGATGAAGTTGC
TGGAGCAGGCAGGTTCTC**TGAAAGGCTCCCTGA
GTGTGGAA**GAGCAGCTGTCACTCATCAGTGGTTG
TCCCAATATCCAAGAAGCAGTAGAGGGTGCCATG
CACATTCAGTAA

The predicted AA sequence (92 aa)

> CRYL1-IFT88 aa

MASSAAGCVVIVGSGVIGRSWAMLFASGGFQ
VKLYDIEQQQIRNALENIRKEMKLLEQAGSLKGSLS
VEEQLSLISGCPNIQEAVEGAMHIQ

The CRYL1-IFT88 function domain predicted by NCBI CD-Search

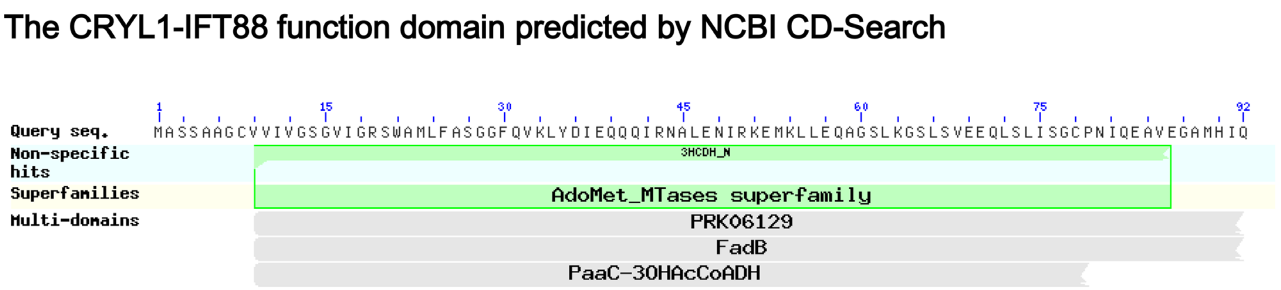

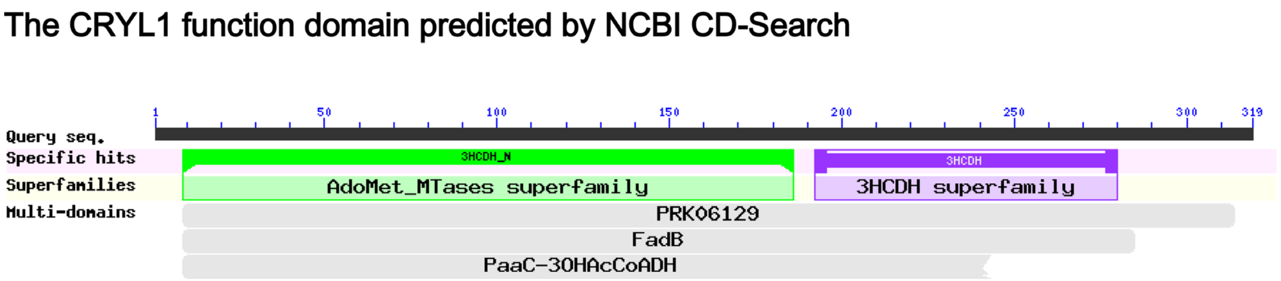

The CRYL1 function domain predicted by NCBI CD-Search

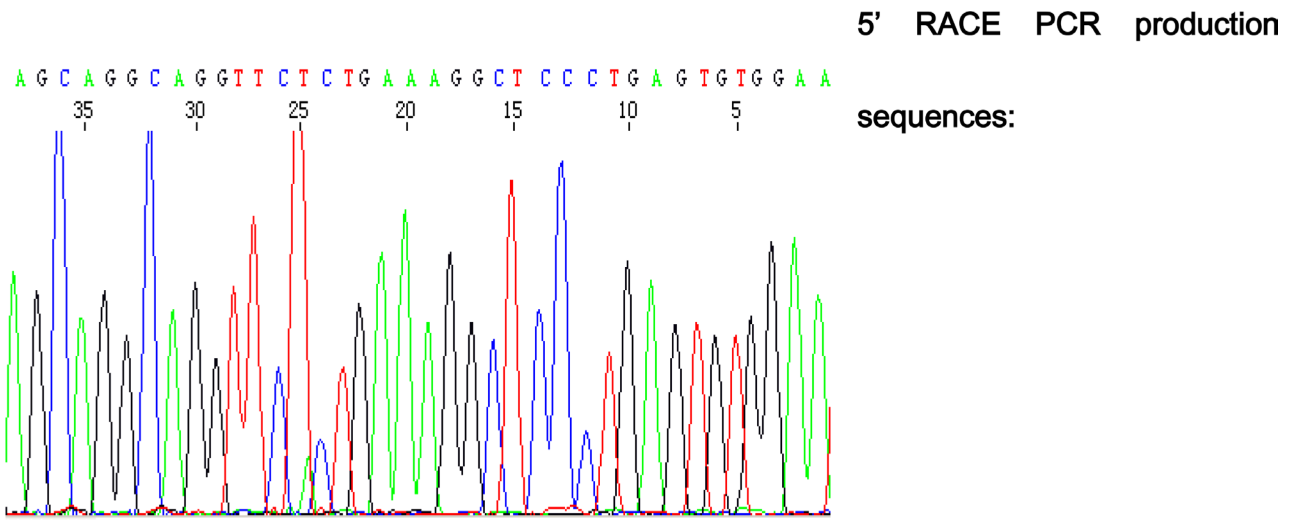

5’ RACE PCR production sequences:

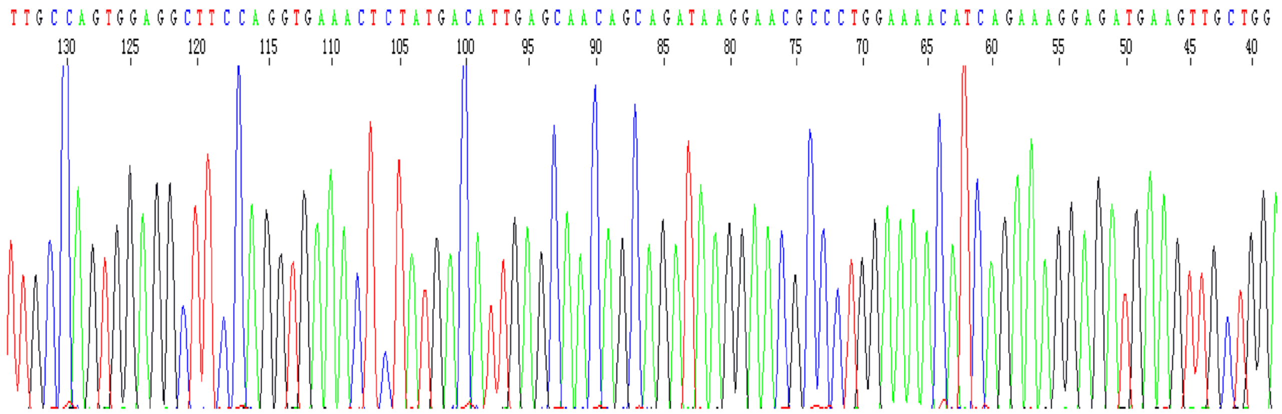

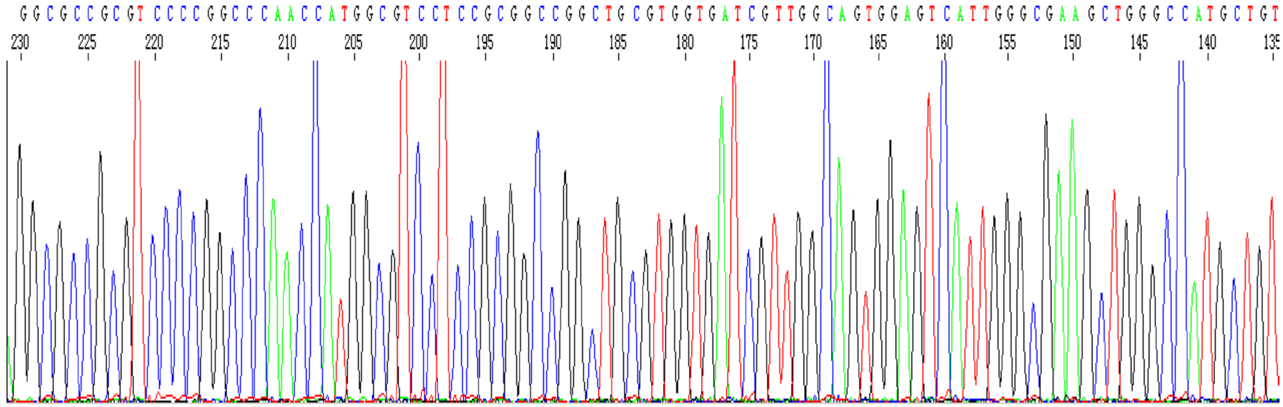

3′ RACE production sequences:

3’ RACE production sequences:

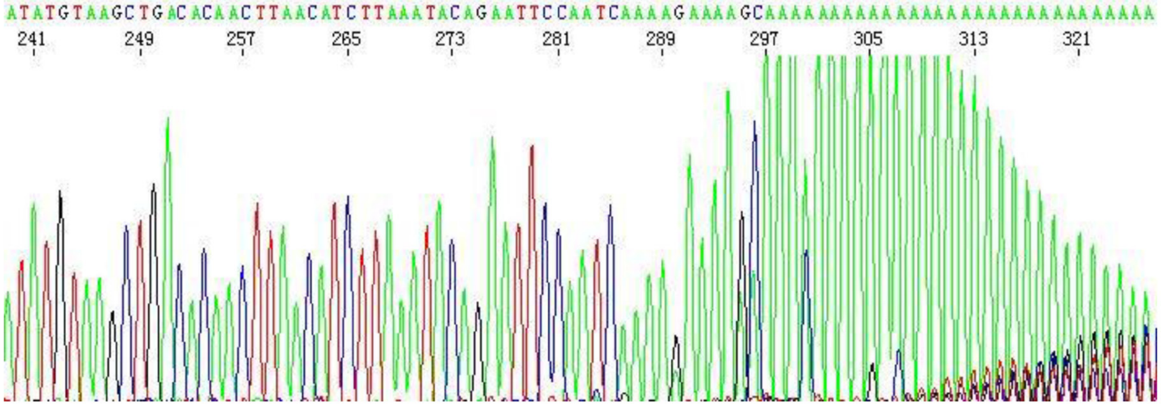

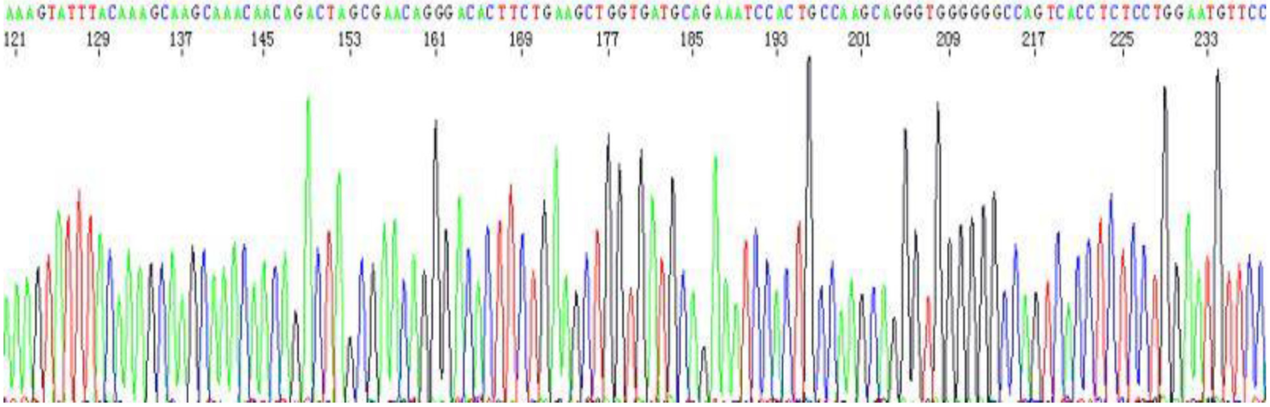

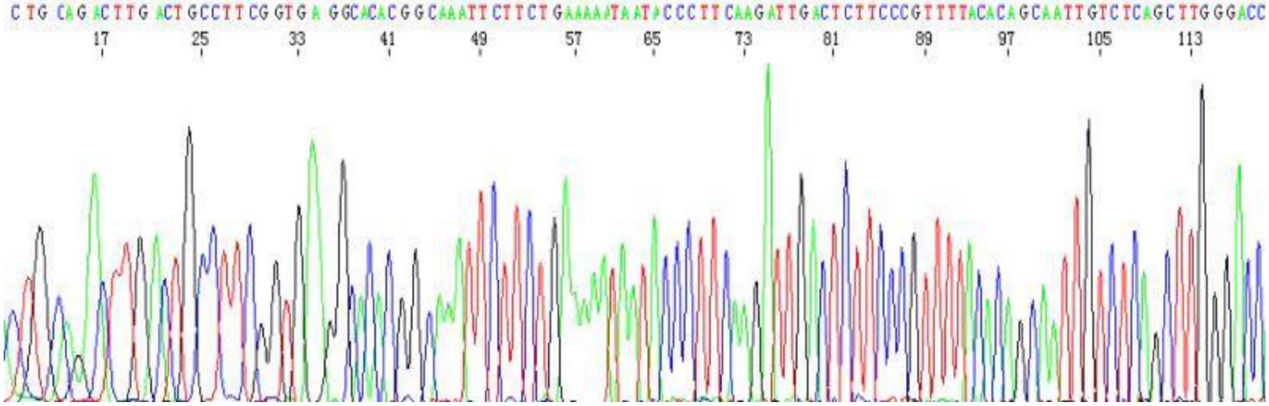

Supplement: Supplementary file 1 [file oncotarget-08-40693-s001.pdf]
